# Huperzine A in the Treatment of Alzheimer's Disease and Vascular Dementia: A Meta-Analysis

**DOI:** 10.1155/2014/363985

**Published:** 2014-02-03

**Authors:** Shu-huai Xing, Chun-xiao Zhu, Rui Zhang, Li An

**Affiliations:** ^1^Department of Nutrition and Food Hygiene, School of Public Health, China Medical University, Shenyang, Liaoning 110001, China; ^2^Shunyi District Centers for Diseases Control and Prevention, Beijing 101300, China

## Abstract

The objective of our study was to perform an updated meta-analysis of placebo-controlled RCTs of Huperzine A (Hup A) on patients with Alzheimer's disease (AD) and vascular dementia (VD), in order to provide the basis and reference for clinical rational drug use. The primary outcome measures assessed were minimental state examination (MMSE) and activities of daily living scale (ADL). Eight AD trials with 733 participants and two VD trials with 92 participants that met our inclusion criteria were identified. The results showed that Hup A could significantly improve the MMSE and ADL score of AD and VD patients, and longer durations would result in better efficacy for the patients with AD. It seemed that there was significant improvement of cognitive function measured by memory quotient (MQ) in patients with AD. Most adverse effects in AD were generally of mild to moderate severity and transient. Compared to the patients with AD, Hup A may offer fewer side effects for participants with VD in this study. Therefore, Hup A is a well-tolerated drug that could significantly improve cognitive performance in patients with AD or VD, but we need to use it with caution in the clinical treatment.

## 1. Introduction

Global population aging has been increasingly evident throughout recent decades, and dementia will become a worldwide problem. Alzheimer's disease (AD) and vascular dementia (VD) are the principal causes of dementia in late life, affecting approximately 10% of people aged at least 65 years worldwide [1]. AD is a devastating, widely distributed, and age-related neurodegenerative disorder and presenting with impaired memory accompanied by a decline in living skills as the main symptom. Currently, the leading approach to symptomatic therapy of AD is based on cholinergic enhancement strategies. Augmentation of cholinergic neurotransmission with the use of cholinesterase inhibitors (ChE-Is) produces a modest improvement in cognitive function for some patients [2, 3]. VD is a type of dementia caused by a variety of cerebral vascular diseases such as cerebral hemorrhage, cerebral infarction, and subarachnoid hemorrhage [4]. More interestingly, cholinergic agents, including ChE-Is, have shown considerable benefits in VD therapy [5]. Therefore, ChE-Is are the standard drugs for treatment of patients with AD and VD.

Huperzine A (Hup A), a new alkaloid and highly reversible ChE-I, is isolated from *Huperzia serrata*. It selectively inhibits acetylcholinesterase activity and thus facilitates the increase in acetylcholine levels in the brain thereby improving cognitive function in patients with dementia. Hup A was first approved for the treatment of AD in China in 1994. Some studies have shown that Hup A induces significant improvement in the memory of elderly people and patients with AD or VD [6–8]. Furthermore, both animal and human safety evaluations have demonstrated that Hup A is devoid of unexpected toxicity [9]. When compared with galantamine, donepezil, tacrine, and so forth, it has longer duration of action, better penetration of the blood-brain barrier, higher oral bioavailability, fewer adverse reactions, and many other advantages [10].

Although many clinical trials have claimed that Hup A appears to offer benefits for some patients with AD or VD without severe adverse effects, a report stated that there was inconclusive evidence showing Hup A to be beneficial for AD or VD [11]. Another review of Hup A for AD [12] concluded that although available trials indicated some benefits from Hup A, the trials were generally small and of limited quality. In addition, only a few are randomized controlled clinical trials with different treatment durations and assessment outcomes. Four recent [1, 13–15] systematic reviews had presented beneficial effect of Hup A on AD or VD patients, but some of them included Chinese articles only, and other studies discussed separately AD or VD. Therefore, the purpose of our study was to perform an updated meta-analysis of placebo-controlled RCTs of Hup A in patients with AD and VD, including clinical trials without language limitation, in order to provide the basis and reference for clinical rational drug use.

## 2. Methods

### 2.1. Search Strategy

English-language electronic searches were performed using Cochrane Library (Jan 1980–May 2013), Medline (Jan 1980–May 2013), and EMBASE (Jan 1980–May 2013) by two authors (Xing and Zhu) independently. At the same time, Chinese-language literatures were searched in the Chinese Biomedical Literature Database, China National Knowledge Infrastructure (Jan 1980–May 2013), and Wanfang database (Jan 1980–May 2013). Keywords were “Huperzine A” (or its trademark names in China such as Ha Bo Yin, Shuang Yi Ping) and “Alzheimer's disease” or “vascular dementia” and the limits were RCTs and human. Recent published reports of clinical trials and review articles identified for inclusion in the meta-analysis were examined to identify additional potentially relevant studies. The investigators (Xing and Zhu) using the Jadad scale extracted the data from the studies meeting the selection criteria.

### 2.2. Inclusion Criteria

#### 2.2.1. AD

Inclusion criteria were as follows: (1) presented original data from a randomized placebo-controlled study; (2) participants with AD and without current diagnosis of any other psychiatric or neurological disorder, aged older than 50 years (all of them were diagnosed on the basis of standardized criteria of Diagnostic and Statistical Manual of Mental Disorders (DSM) III, III-R, and IV or the National Institute of Neurological and Communicative Disorders and Stroke-Alzheimer's Disease and Related Disorders Association Criteria (NIDCDS/ADRDA) [16]); (3) outcome measures of cognitive performance MMSE and ADL in AD patients; (4) having a minimum treatment duration of 6 months, a minimum number of participants of five per group, and the availability of a full-text publication.

#### 2.2.2. VD

Inclusion criteria were as follows: (1) presented original data from a randomized placebo-controlled study; (2) participants with VD and without current diagnosis of any other psychiatric or neurological disorder (all of patients were diagnosed on the basis of standardized criteria of DSM IV and IV-R or National Institute of Neurological and Communicative Disorders and Stroke and Association Internationale pour la Recherche et L'Enseignement en Neuroscience (NINCD/AIREN) [17]; (3) MMSE and ADL performed to detect the cognitive performance of participants; (4) evidence of cerebrovascular disease in brain imaging (CT or MRI); (5) having a minimum treatment duration of 12 weeks, a minimum number of participants of five per group, and the availability of a full-text publication.

### 2.3. Exclusion Criteria

Enclusion criteria were as follows: (1) AD or VD trials with fewer than 10 participants in each arm; (2) patients with specific types of non-Alzheimer's dementia (non-AD) or nonvascular dementia (non-VD), such as Lewy-body dementia or dementia due to Parkinson's disease.

### 2.4. Data Extraction

Two independent investigators (Xing and Zhu) extracted data from the collected reports, and disagreements were resolved by discussion with another investigator. The following data were documented from each trial: trial name, publication year, number of participants, sample size, diagnosis criteria, primary variable, and treatment regimen. The primary analysis was to compare Hup A versus placebo based on MMSE and ADL assessment. All the endpoints outcome data in each trial were chosen for the meta-analysis.

### 2.5. Statistical Analysis Methods

Statistical analysis was performed by RevMan 5.0 software [18]. Mean difference in the changes of mean score from baseline between Hup A group and placebo group was used to evaluate Hup A curative effects. Test of heterogeneity was assessed using the *I* ^2^ test, with *I* ^2^ quantifying the proportion of the total outcome variability attributable to variability among the trials. *I* ^2^ of at least 50% were taken as indicators of substantial heterogeneity of outcomes. Homogenous data was calculated using the fixed-effect model, and random-effect model was employed when there was statistically significant heterogeneity. Considering that heterogeneity in treatments could be related to the duration of trial, thus subgroup analysis was used to explore possible sources of heterogeneity. Sensitivity analysis was performed by excluding the trials which potentially biased the results, and the stability of outcome was tested by sensitivity analysis when necessary. Adverse effects were tabulated and assessed with descriptive techniques. The possible publication bias was assessed by visual asymmetry of a funnel plot and the fail-safe N_0.05_(Nfs_0.05_). Nfs_0.05_ = *K*(*Z* ^2^ − 1.645^2^)/1.645^2^. Statistical data was expressed as 95% confidence interval and with *P* < 0.05 for the difference was statistically significant.

## 3. Results

### 3.1. Literature Search

#### 3.1.1. AD

**Figure 1:**
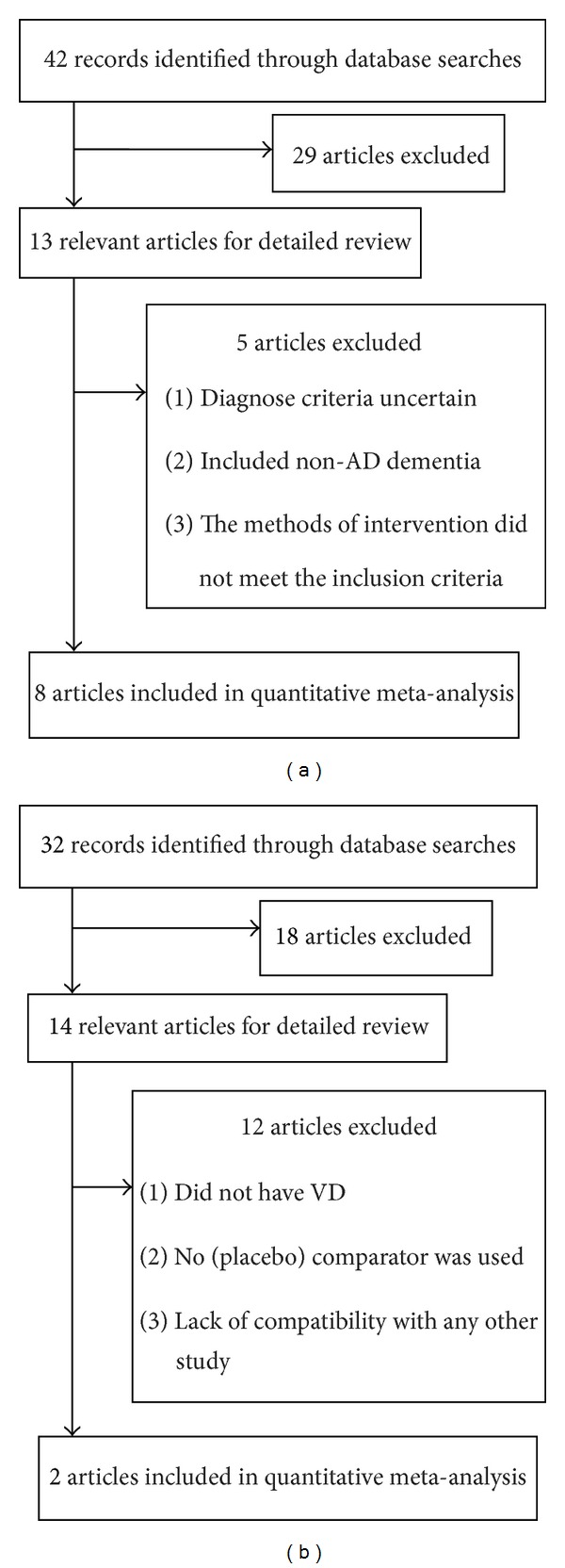
Flow diagram of the study selecting process for AD (a) and VD (b).

The search strategy identified forty-two potential studies from the databases (Figure 1(a)). Twenty-nine of these articles were excluded according to our inclusion criteria because they were clearly irrelevant to the objectives of our meta-analysis. One trial [19] was excluded because the AD patients were not diagnosed with AD by DSM or NINCDS/ADRDA criteria. Two trails [20, 21] were also excluded for including non-AD dementia. Two positive controlled clinical trials [6, 22] were excluded because the types of intervention did not meet the inclusion criteria. Finally, eight trials were included in the meta-analysis based on our inclusion criteria. A total of 733 participants were included in the eight studies, with 360 in the Hup A group and 373 in the control group. The number of patients in the individual studies ranged from 28 to 197, and the durations of trial ranged from 8 to 24 weeks.

#### 3.1.2. VD

Thirty-two potential studies were identified which met the search strategy (Figure 1(b)). Eighteen of these articles were excluded according to our inclusion criteria because they were clearly irrelevant to the objectives of our meta-analysis. In addition, the following trials were excluded: in four trails [23–26], the participants did not have VD; seven studies [27–33] were open-label; that is, no (placebo) comparator was used; one study [34] with data could not be included in the meta-analysis because of a lack of compatibility with any other study. At last, two trials were included in the meta-analysis based on our inclusion criteria. A total of 92 participants were included in the two studies, with 46 in the Hup A group and 46 in the control group. The number of patients in the individual studies ranged from 14 to 78, and the durations of trial ranged from 12 to 24 weeks.

### 3.2. Study Quality Assessment and Treatment Regimen

#### 3.2.1. AD

The Jadad quality scale was used for methodological quality assessment of each trial and a total score was computed by summing the scores of all criteria (range: 0–5). Four studies had a Jadad quality score greater than 4 and the median score was 3.75. All selected trials were randomized. Three trials were single-blind [35–37], and the other five trials [38–42] were double-blind. Two trials [36, 39] reported the explanation of withdrawing patients and only one trial [39] used the full analysis set based on the intent-to-treat principle. The description of the trial characteristics and demographics of the participants in the studies were shown in Table 1.

Patients in the Hup A group received Hup A tablets orally for 8–24 consecutive weeks. At the same time, blank tablets were supplied to the patients in the control group, except those in the study [36] who received *Salvia miltiorrhiza* tablets. In one trial [39], all participants received vitamin E (100 mg/day) as routine treatment.

#### 3.2.2. VD

The Jadad quality scale was used for methodological quality assessment of each trial and a total score was computed by summing the scores of all criteria (range: 0–5). Both of the two studies had a Jadad quality score greater than 3 and the median score was 3.5. All selected trials were randomized. One trial [43] was single-blind, and the other [4] was double-blind. The description of the trial characteristics and demographics of the participants in the studies were also showed in Table 1.

Patients in the treatment group were treated orally with Hup A. Patients received Hup A tablets or placebo orally for 12–24 consecutive weeks. At the same time, blank tablets were supplied to the patients in the control group. In one trial [4], patients in control were treated orally with 100 mg of vitamin C bid.

### 3.3. Meta-Analysis

#### 3.3.1. AD

**Figure 2:**
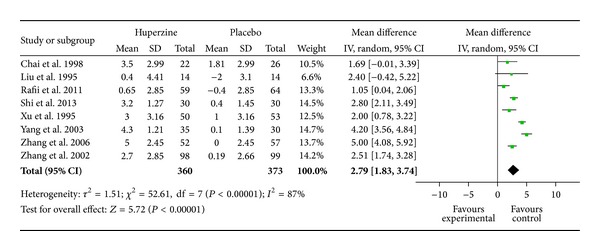
Forest plot with the weighted mean difference (WMD) on minimental state examination (MMSE) of Hup A relative to placebo in AD with 95% CI of the trials included in meta-analysis.

Figure 2 showed the comparison of change of MMSE scores between Hup A and placebo groups. There was a significant amount of heterogeneity (*I* ^2^ = 87%, *P* < 0.00001); thus random-effect model was used to estimate the pooled effect size. There was a beneficial effect of Hup A in the improvement of general cognitive function for AD (WMD: 2.79, 95% CI, 1.83 ~ 3.74, *P* < 0.00001). To further explore heterogeneity, we performed subgroup analysis by treatment duration (6 weeks, 8 weeks, 12 weeks, and 16 weeks). It was shown that Hup A was superior to placebo in the improvement of MMSE at 6 weeks (WMD: 1.56; 95% CI, 0.68 ~ 2.44, *P* = 0.0005), 8 weeks (WMD: 1.95; 95% CI, 1.01 ~ 2.89,  *P* < 0.0001), 12 weeks (WMD: 1.96; 95% CI, 0.66 ~ 3.25, *P* = 0.003), and 16 weeks (WMD: 2.79; 95% CI, 1.05 ~ 4.54, *P* = 0.002). Interestingly, the pooled effect increased gradually with the prolongation of the treatment duration. When taking the sensitivity analysis, we found an article [41] that interferes the overall result very much and has heterogeneity to other studies. But when we removed this study, the heterogeneity still did not disappear and the result did not change obviously (data not shown). When omitting one study in each turn, we found that the pooled effect ranges from 2.46 to 2.92 and the *I* ^2^ value ranges from 82% to 89%. The data above has indicated that the main result was robustness. A funnel plot revealed a significantly less asymmetrical distribution of studies for MMSE (Figure 4(a)), and the Nfs_0.05_ was 11. These results showed that the possibility of publication bias was large.

**Figure 3:**
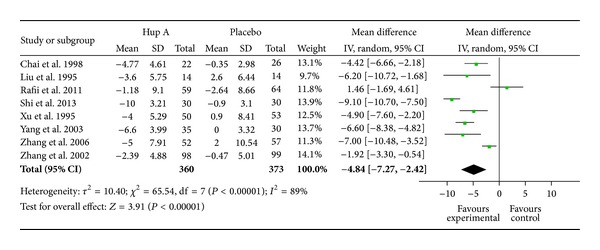
Forest plot with the weighted mean difference (WMD) on daily living scale (ADL) of Hup A relative to placebo in AD with 95% CI of the trials included in meta-analysis.

**Figure 4:**
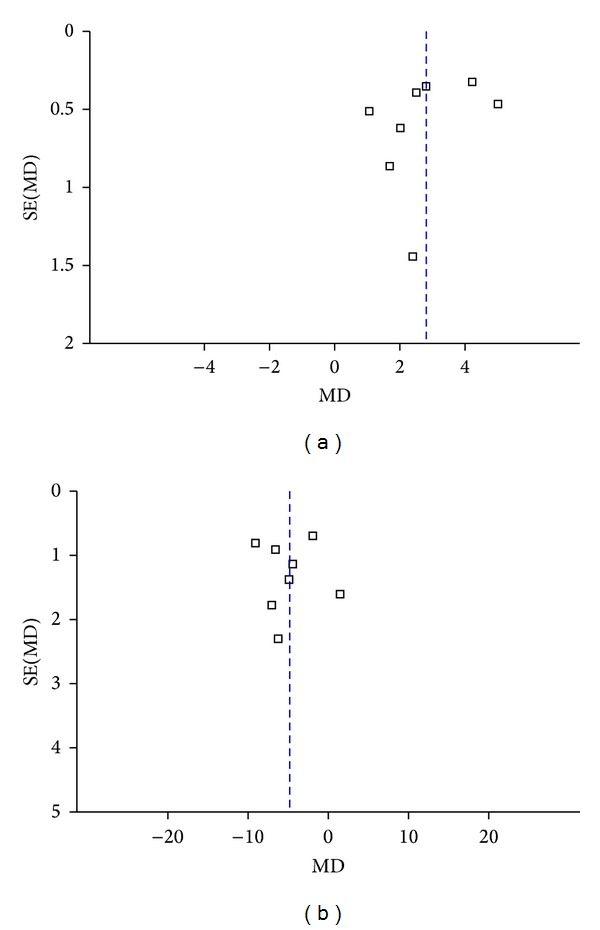
Funnel plot of comparison for AD: Hup A versus placebo; outcome: MMSE (a) and ADL (b).

Figure 3 showed the mean difference in the changes of ADL score from baseline between Hup A and placebo groups. The pooled effect size was −4.84 (95% CI, −7.27 ~ −2.42,  *P* < 0.0001). A negative value indicated an improvement in condition. The random effect model was used because of a significant amount of heterogeneity (*I* ^2^ = 89% *P* < 0.00001). The difference of durations (6 weeks, 8 weeks, 12 weeks, and 16 weeks) in the treatment regimens best explains the high heterogeneity among the studies. Therefore, we stratified trials in 4 subgroups according to the duration of treatment regimens. The results of our study indicated that Hup A was superior to placebo in the improvement of ADL at 6 weeks (WMD: −2.36; 95% CI, −3.68 ~ −1.04, *P* = 0.0005), 8 weeks (WMD: −4.82; 95% CI, −6.43 ~ −3.21, *P* < 0.00001), 12 weeks (WMD: −5.50; 95% CI, −12.53 ~ 1.54, *P* = 0.13), and 16 weeks (WMD: −6.60; 95% CI, −8.38 ~ −4.82, *P* < 0.00001). Although there was no significantly statistical difference between two groups at 12 weeks (*P* = 0.13), generally, it showed that longer duration would result in better efficacy. When taking the sensitivity analysis, we found an article [40] that interferes the overall result very much and has heterogeneity to other studies. When we removed this study, the heterogeneity decreased and the result changed greatly (the pooled effect was −5.68), so the study was notable and distinctive. Then a single study involved in the meta-analysis was deleted each time to reflect the influence of the individual data set on the pooled effects, and the corresponding pooled effects were not materially altered (data not shown). Visual inspection of the funnel plot (Figure 4(b)) and the Nfs_0.05_  (Nfs_0.05_ = 37) showed that evidence of publication bias existed in these studies, but the publication bias was not large.

Weighted mean difference on Hasegawa dementia scale (HDS) of Hup A relative to placebo in AD was shown in Table 2. The pooled effect size was 2.80 (95% CI, 1.12 ~ 4.49,  *P* = 0.001) indicating a beneficial effect of Hup A. The significant amount of heterogeneity (*I* ^2^ = 83%, *P* = 0.0007) also existed, so the random effect model was used. Interestingly, when we removed the study [41], the heterogeneity decreased but the result changed slightly (data not shown). With all four included studies, no funnel plot asymmetry was found, and the Nfs_0.05_ was 12, indicating that the result was less affected by publication bias. Table 2 showed AD assessment scale-cognitive subscale (ADAS-Cog) of Hup A relative to placebo in AD. Since heterogeneities existed between these studies (*I* ^2^ = 82%, *P* = 0.02), the random effect model was used. The two trials pooled effect size was −3.01 (95% CI, −8.24 ~ −2.22, *P* = 0.26), suggesting no significant difference between two groups. A funnel plot asymmetry was found (data not shown). Compared to the number of selected literatures, the publication bias existed (Nfs_0.05_ = −1). Table 2 showed the comparison of change of memory quotient (MQ) scores between Hup A and placebo groups. Significant evidence of heterogeneity between trials was not observed (*I* ^2^ = 1%, *P* = 0.39); thus a fixed-effects model provided the same overall effect. And there was a statistical superiority for Hup A compared to placebo (WMD = 7.44, 95% CI: 4.70 ~ 10.19, *P* < 0.00001). The results of this meta-analysis indicated that administration of Hup A leads to a significant improvement in MQ of patients with AD. A funnel plot analysis was symmetrical on the whole (data not shown) and the Nfs_0.05_ was 38, demonstrating no significant publication bias.

#### 3.3.2. VD

There was an improvement of 4.92 points (95% CI: 1.80 ~ 8.04, *P* = 0.002) in the MMSE for the Hup A group compared to the placebo group (Table 2). However, heterogeneity was substantial (*I* ^2^ = 63%, *P* = 0.10), so the random-effect model was used to estimate the pooled effect size. There were statistically significant differences in ADL change scores between Hup A and placebo, with WMD = −10.24 (95% CI: −16.66 ~ −3.83, *P* = 0.002) (Table 2). Again, heterogeneity was substantial (*I* ^2^ = 81%, *P* = 0.02), so the random-effect model was also used to estimate the pooled effect size. An examination of the funnel plot for our data suggests strong evidence of publication bias for MMSE and ADL in the meta-analysis (data not shown). Analogously, the Nfs_0.05_ both of them were 5, so more large-sample, high-quality randomized studies are needed.

### 3.4. Safety and Tolerability—Incidence of Adverse Events

#### 3.4.1. AD

**Figure 5:**
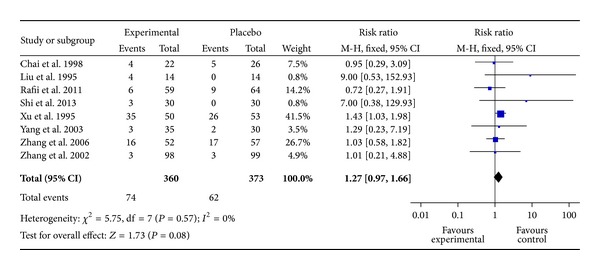
Number of cases with side effects in Hup A group versus placebo group for AD.

Figure 5 showed the number of cases with side effects in Hup A group versus placebo group. Significant evidence of heterogeneity between trials was not observed (*I* ^2^ = 0%, *P* = 0.57), so the fixed-effects model was used to provide the pooled effect. There was no significant difference between two groups (RR = 1.27, 95% CI: 0.97 ~ 1.66,  *P* > 0.05). Of those adverse effects, some mild peripheral cholinergic side effects, such as dry mouth, mild bellyache, and diarrhea, were more likely to occur in the Hup A group than in the placebo group (Table 3). No adverse effects on vital signs, blood test results, or electrocardiogram results were observed. But it was shown that there was significant difference in nausea or vomiting between two groups (OR 3.20, 95% CI: 1.15 ~ 8.90 *P* < 0.05).

#### 3.4.2. VD

The most frequently observed adverse effects were gastrointestinal upset or constipation with no significant difference between two groups. Only one patient in the Hup A group experienced mild nausea and dizziness. These symptoms resolved by themselves and did not affect continuation of the treatment.

## 4. Discussion

### 4.1. AD

Studies on dementia treatment thus far have yielded findings that suggest modest benefits using ChE-I in patients with AD based on the effect sizes. The results from our meta-analysis of eight randomized controlled trials showed that Hup A could significantly improve the MMSE and ADL score of AD patients. Although the study [39] might be a confounded study, it is reported that there was no evidence of the efficacy of vitamin E for people suffering from AD and mild cognitive impairment [44]. Besides, in a trial [36], participants in the control received *Salvia miltiorrhiza* tablets, but there is no convincing evidence regarding its positive efficacy. Therefore, it was considered as a placebo in our analysis. The significant amount of heterogeneity existed among the studies. There were some obvious differences among the eight studies, such as the severity of AD and mean MMSE scores before treatment and publication year, as well as the duration of Hup A and mean age, all of which might contribute to heterogeneity among the trials. From the information available, we performed prespecified subgroup analyses comparing patients with different durations for each outcome. The treatment difference of both MMSE and ADL on treatment duration showed that longer duration would result in better efficacy. For MMSE, sensitivity analysis by omitting individual studies supported that the overall result was robustness. Through the sensitivity analysis for ADL, however, we found a trial [40] which was different from others, since this study was conducted in USA and the possible influence factors may include experimental method, gene polymorphism, different races, and so forth. For the two outcome mentioned above, the publication bias was unavoidable if the related study cannot be collected as required. It was possible to increase more large-sample, high-quality randomized studies to assess the bias. In our opinion, although the English trial was distinctive, we can still consider that the treatment effect improved gradually with the prolongation of the treatment duration.

In addition, it seemed that there was significant improvement of cognitive function measured by MQ. The main result was reliable and showed robustness. In the other study by Sun et al. [45], the MQ scores between the placebo and Hup A groups were statistically significant too. Moreover, the results showed that there was significant improvement of cognitive function measured by HDS. But the significant amount of heterogeneity existed in HDS. One trial [41] was across the midline in the Forest plot. The sensitivity analysis on the comparison of HDS score changes showed that the influence of the quality of the RCTs could reverse the results. Larger high-quality trials would be needed to detect effects with any reliability. Results showed that ADAS-Cog as an outcome measure did not reach statistical significance. In this study, ADAS-cog acted as an outcome measure only in 2 trials, and there was significant difference between them. The result was due to the limited amount of data of the included studies. Thus, more evidence is needed to reach reliable conclusion.

Currently, there is insufficient data available to determine the toxicity of Hup A in humans, although only mild to moderate cholinergic side effects have been reported at therapeutic doses [46]. Hup A was shown to be well-tolerated; however, the results of our meta-analysis showed that there was significant difference in nausea or vomiting between two groups. Most adverse effects were related to cholinergic activity of this class of drug, but most of them were generally of mild to moderate severity and transient. In a word, Hup A was a well-tolerated drug for AD, but we need to use it with caution in the clinical treatment.

### 4.2. VD

In the whole trial population, statistically significant treatment effects in favor of Hup A were compared with placebo in cognition. We observed that the MMSE and ADL scores of patients with VD significantly improved after 12 weeks of treatment with Hup A. But our results need to be explained with caution because of the study of Xu et al. [4] using vitamin C as placebo. It is reported that Vitamin C is probably beneficial for VD [47]. Our sample size was so small that the results are uncertain and not showed robustness. Since it was unclear whether most trials had used positive drugs as control, compared with others trials, we had to select the two more suitable trials. In two trials, the confidence interval for the treatment effect estimate was wide, and it included both clinically significant benefits and clinically significant harms. In addition, our analysis included only two trials with 92 participants, and one of them was single-blinded, so more high-quality, large sample, randomized placebo-controlled studies are needed to determine whether there is worthwhile effect for VD. The insufficient number of studies prohibited us from meaningful sensitivity analysis to illuminate how robust the results of the study are.

In the two trials, we found that only one patient experienced mild nausea and dizziness. It was shown that Hup A was well-tolerated for VD, but as it just existed in our analysis, larger and better quality evidence is required.

## 5. Conclusions

The meta-analysis of Hup A reported here highlights that this treatment has certain significant improvement for patients with AD or VD, and longer durations may result in better efficacy for patients with AD. Compared to the patients with AD, we found that Hup A may offer fewer side effects for participants with VD in this study, which might exist as an accidental phenomenon because of the insufficient number of studies for VD. Compared with other previous meta-analyses, we increased the number of trails and more outcomes to determine the effect of Hup A. Besides, we added the patients with VD to review the role of Hup A in the treatment of VD. But there are some limitations that existed in the analysis. At first, the number of VD studies is quite small. Secondly, there might be a publication bias in this review, and the possible reason was publishing of unusually high proportions of positive results [48]. Thirdly, it was because of lack of data in the RCTs on quality of life and caregiver burden that we were not able to draw conclusions about these important outcomes. Furthermore, conference papers were not included in our study because most of the full text was unavailable, so there might exist fugitive literatures. Finally, most of the selected trials had no intention-to-treat analysis. Therefore, if we want to research further studies, all of the above need to be considered.

## Figures and Tables

**Table 1:** Study characteristics of all included clinical trials (AD and VD).

Study	Publication year	Patient population	Sample size	Diagnosis criteria	Primary variable	Treatment regimen	Jadad quality score
AD							
Shi et al. [37]	2013	Age ≥ 50, MMSE ≤ 26	60	DSM4	MQ, MMSE, HDS-R, ADL	400 *μ*g/day, 12 weeks	3
Rafii et al. [40]	2011	Age ≥ 50, MMSE (range): 10–24	123	NINCDS/ADRDA	ADAS-Cog, MMSE, NPI, ADCS-ADL	200 *μ*g/day, 16 weeks	5
Zhang et al. [36]	2006	Age (range): 52–78, MMSE (mean ± SD): 15 ± 4	109	DSM3-R	MMSE, ADL	500 *μ*g/day, 24 weeks	3
Yang et al. [35]	2003	Age (range): 65–90, MMSE ≤ 26	65	DSM4	MMSE, CDR, ADL	300 *μ*g/day, 16 weeks	3
Zhang et al. [39]	2002	Age (range): 50–80, MMSE (range): 10–26	197	DSM4	ADAS-cog, MMSE, ADL, ADAS-non-Cog, CIBIC plus	400 *μ*g/day, 12 weeks	5
Chai et al. [42]	1998	Age (range): 40–62, MMSE (range): 13–23, ADL > 20, HDS (range): 10–30,	48	DSM3-R	MQ, MMSE, HDS, ADL	400 *μ*g/day, 8 weeks	4
Xu et al. [38]	1995	Age > 50, MMSE (range): 13–23	103	DSM3-R	MMSE, HDS, ADL, MQ	400 *μ*g/day, 8 weeks	3
Liu et al. [41]	1995	Age (range): 51–92, MMSE (range): 10–26,	28	DSM3-R	MMSE, HDS, ADL, MQ	200 *μ*g/day, 8 weeks	4
VD							
Xu et al. [4]	2012	Age (range): 60–85, MMSE (range): 12–24	78	DSM4-R NINDS-AIREN	MMSE, CDR, ADL	100 *μ*g/day, 12 weeks	4
Zhou et al. [43]	2004	Age (mean ± SD): 70.25 ± 10.89, MMSE (mean ± SD): 13.4 ± 5.2	14	DSM4	MMSE, ADL	300 *μ*g/day, 24 weeks	3

**Table 2:** Other results of meta-analysis for the efficacy of Hup A for AD or VD patients.

Parameters	*N*	Sample size (H/P)	Heterogeneity	Pooled effect	*Z* Test
AD					
HDS	4	116/123	*χ* ^2^ = 17.17, *P* = 0.0007, *I* ^2^ = 83%	2.80 [1.12, 4.49]	*Z* = 3.26, *P* = 0.001
ADAS-Cog	2	157/160	*χ* ^2^ = 5.58, *P* = 0.02, *I* ^2^ = 82%	−3.01 [−8.24, 2.22]	*Z* = 1.13, *P* = 0.26
MQ	4	116/123	*χ* ^2^ = 3.03, *P* = 0.39, *I* ^2^ = 1%	7.44 [4.70, 10.19]	*Z* = 5.31, *P* < 0.00001
VD					
MMSE	2	46/46	*χ* ^2^ = 2.69, *P* = 0.10, *I* ^2^ = 63%	4.92 [1.80, 8.04]	*Z* = 3.09, *P* = 0.002
ADL	2	46/46	*χ* ^2^ = 5.21, *P* = 0.02, *I* ^2^ = 81%	−10.24 [−16.66, −3.83]	*Z* = 3.13, *P* = 0.002

**Table 3:** Incidence of adverse events for AD.

Adverse event	Number of subjects (pooled occurrence)		Odd ratio (fixed) 95% CI
	Huperzine A (*n* = 360)	Placebo (*n* = 373)	
Agitation	3 (0.83)	3 (0.80)	1.04 [0.21, 5.17]
Ankles edema	1 (0.28)	0 (0.00)	3.12 [0.13, 76.76]
Anorexia	12 (3.33)	6 (1.60)	2.11 [0.78, 5.68]
Bradycardia	0 (0.00)	1 (0.27)	0.34 [0.01, 8.48]
Constipation	4 (1.11)	0 (0.00)	9.43 [0.51, 175.77]
Diarrhea	5 (1.39)	2 (0.54)	2.61 [0.50, 13.55]
Dizziness	9 (2.5)	12 (3.21)	0.77 [0.32, 1.85]
Dry mouth	4 (1.11)	0 (0.00)	9.43 [0.51, 175.77]
Diaphoresis	4 (1.11)	0 (0.00)	9.43 [0.51, 175.77]
Dimness of vision	0 (0.00)	1 (0.27)	0.34 [0.01, 8.48]
Festinating gait	0 (0.00)	1 (0.27)	0.34 [0.01, 8.48]
Headache	2 (0.56)	4 (1.07)	0.52 [0.09, 2.83]
Hyperactivity	5 (1.39)	3 (0.80)	1.74 [0.41, 7.32]
Hypopraxia	0 (0.00)	1 (0.27)	0.34 [0.01, 8.48]
Hypersomnia	4 (1.11)	0 (0.00)	9.43 [0.51, 175.77]
Indigestion	5 (1.39)	2 (0.54)	2.61 [0.50, 13.55]
Insomnia	6 (1.67)	6 (1.60)	1.04 [0.33, 3.24]
Mild bellyache	6 (1.67)	0 (0.00)	13.70 [0.77, 244.02]
Nasal obstruction	4 (1.11)	4 (1.07)	1.04 [0.26, 4.18]
Nausea or vomiting	15 (4.16)	5 (1.34)	3.20 [1.15, 8.90]
